# The hepcidin-ferroportin axis controls the iron content of *Salmonella*-containing vacuoles in macrophages

**DOI:** 10.1038/s41467-018-04446-8

**Published:** 2018-05-29

**Authors:** Daejin Lim, Kwang Soo Kim, Jae-Ho Jeong, Oriana Marques, Hyun-Ju kim, Miryoung Song, Tae-Hoon Lee, Jae Il Kim, Hueng-Sik Choi, Jung-Joon Min, Dirk Bumann, Martina U. Muckenthaler, Hyon E. Choy

**Affiliations:** 1 Department of Microbiology, Chonnam National University Medical School, Gwangju, 61468 Republic of Korea; 2 Department of Molecular Medicine (BK21plus), Chonnam National University Graduate School, Gwangju, 61468 Republic of Korea; 3 Department of Pediatric Hematology, Oncology and Immunology - University of Heidelberg, Im Neuenheimer Feld 350, Heidelberg, D-69120 Germany; 4 Molecular Medicine Partnership Unit, Heidelberg, D-69120 Germany; 5 Translational Lung Research Center Heidelberg (TLRC), German Center for Lung Research (DZL), University of Heidelberg, Heidelberg, D-69120 Germany; 6 Department of Biochemistry, Dental Science Research Institute, School of Dentistry, Chonnam National University and Korea mouse phenotype center (KMPC), Gwangju, 61186 Republic of Korea; 7 School of Life Sciences, Gwangju Institute of Science and Technology, Gwangju, 61005 Republic of Korea; 8 AnyGen, Gwangju Technopark, Gwangju, 61008 Republic of Korea; 9 National Creative Research Initiatives Center for Nuclear Receptor Signals and Hormone Research Center, School of Biological Sciences and Technology, Chonnam National University, Gwangju, 61186 Republic of Korea; 10 Department of Nuclear Medicine, Chonnam National University Medical School, Gwangju, 61469 Republic of Korea; 11 Focal Area Infection Biology, University of Basel, Basel, CH-4056 Switzerland

## Abstract

Macrophages release iron into the bloodstream via a membrane-bound iron export protein, ferroportin (FPN). The hepatic iron-regulatory hormone hepcidin controls FPN internalization and degradation in response to bacterial infection. *Salmonella typhimurium* can invade macrophages and proliferate in the *Salmonella*-containing vacuole (SCV). Hepcidin is reported to increase the mortality of *Salmonella*-infected animals by increasing the bacterial load in macrophages. Here we assess the iron levels and find that hepcidin increases iron content in the cytosol but decreases it in the SCV through FPN on the SCV membrane. Loss-of-FPN from the SCV via the action of hepcidin impairs the generation of bactericidal reactive oxygen species (ROS) as the iron content decreases. We conclude that FPN is required to provide sufficient iron to the SCV, where iron serves as a cofactor for the generation of antimicrobial ROS rather than as a nutrient for *Salmonella*.

## Abstract (web summary)

The effects of iron on vacuole-resident *Salmonella* in macrophages are unclear. Here the authors show that the bacteria are not subject to nutritional inhibition by iron deprivation, but that iron depletion in the vacuole, via the hepcidin-ferroportin axis, inhibits the bactericidal effect of oxidative burst.

## Introduction

In response to infection, mammalian hosts deploy a strategy to withhold iron, an essential trace element for bacterial proliferation^1^. This process is a central component of innate nutritional immunity controlled by the master iron regulatory hormone hepcidin, which is synthesized primarily in the liver in response to interleukin-6 (IL-6) signaling upon bacterial infection^2,3^. Hepcidin promotes the degradation of its receptor, the sole known cellular iron exporter ferroportin (FPN), resulting in iron retention by macrophages and reduction of intestinal iron absorption^2^. This mechanism decreases the serum iron content to about 30% of its normal level, a physiological change known as hypoferremia of infection^4^, which limits iron availability for extracellular bacteria but also leads to increased iron storage in macrophages of the liver and spleen. Previously, we reported that induction of hepcidin and eventual hypoferremia upon *Salmonella* infection was mediated by estrogen-related receptor (ERR)γ^5^. An inverse agonist of ERRγ (GSK5182) ameliorated *Salmonella*-mediated hypoferremia through reduction of hepcidin expression. Treatment of mice infected with *Salmonella* with GSK5182 resulted in an ~30% decrease of iron in hepatic macrophages and a 50–100-fold decrease in bacterial cell numbers in the liver and spleen, resulting in increased survival of the mice. This was interpreted as a typical representation of innate nutritional immunity, under the assumption that the iron status in the vacuoles in which *Salmonella* reside (SCV)^6^ would be the same as in the macrophage cytosol.

In this study, we used a reporter system to assess iron and reactive oxygen species (ROS) levels in the SCV, and demonstrated that hepcidin affected ROS generation in macrophages by modulating iron levels in the SCV. This effect was dependent on the presence of FPN in the SCV.

## Results

### FPN associates with the SCV membrane in absence of hepcidin

**Fig. 1:**
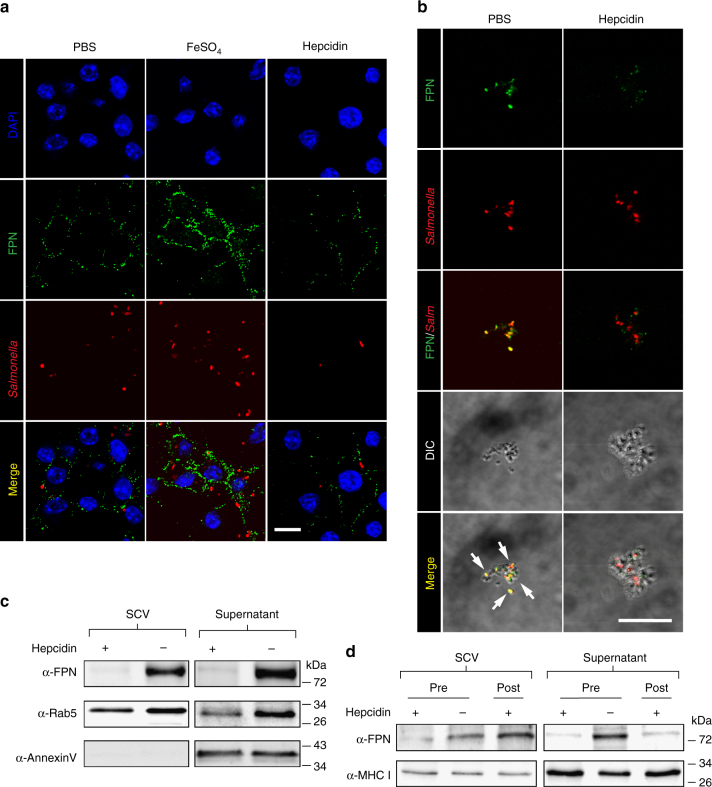
The presence of FPN in the SCV of Raw264.7 cells. **a** Raw264.7 cells pretreated with FeSO_4_ (0.1 mM) and/or hepcidin (1 μg/ml) for 3 h were infected with *Salmonella* (MOI, 100) and examined by confocal microscopy. Enlarged images of merge panels are shown in Supplementary Fig. 17. **b** The cells pretreated with PBS or hepcidin were infected with magnetized *Salmonella* (MOI, 100) for 2 h, and then SCV were isolated and *Salmonella* were analyzed by confocal microscopy. Staining for FPN is shown in green, and *Salmonella* in red. The isolated SCV were visualized by differential interphase contrast (DIC) imaging (scale bar 10 μm). Images are representatives of over 500 isolated SCVs per group from at least three independent experiments. **c** Raw264.7 cells pretreated with hepcidin were infected with magnetized *Salmonella*. FPN, Rab5, and annexin V in the SCV and the non-SCV fraction (supernatant) were examined by western blotting using specific antibodies. **d** Alternatively, Raw264.7 cells infected with *Salmonella* were treated with hepcidin for 2 h post infection and analyzed as in **c**

*Salmonella* enters epithelial cells and macrophages by micropinocytosis and resides in unique membrane-bound compartments SCV^6^. Raw264.7 cells were pretreated with FeSO_4_ to induce FPN expression^7^ or with hepcidin to induce FPN degradation^3^ and then infected with *Salmonella* for 2 h (Fig. 1a). Confocal microscopy confirmed an increase in FPN with FeSO_4_ treatment and a reduction in FPN with hepcidin treatment. *Salmonella* were located immediately adjacent to FPN in cells treated with FeSO_4_, suggesting that FPN is associated with the SCV (see merged images). FPN remained with the SCV even at 12 h after infection in cells treated with FeSO_4_ (Supplementary Fig. 1). To further verify the above observation, SCV were isolated from phagocytic cells using magnetized bacteria^8^. Raw264.7 cells were pretreated with hepcidin, and then infected with *Salmonella* coated with magnetite nanoparticles, which does not alter total FPN levels in the cell (Supplementary Fig. 2). At 2 h post infection (p.i.), the cells were fractionated, and SCV containing magnetized *Salmonella* were separated on a magnet. Initially, we analyzed the SCV fraction and the supernatant for the expression of the macrophage specific membrane marker, F4/80^9^, and observed that it was only detected in the supernatant fraction, suggesting that the isolated SCV was free of most membrane components (Supplementary Fig. 3). We next probed the SCV for the presence of FPN and *Salmonella* by confocal microscopy using specific antibodies (Fig. 1b). FPN was detected only in the SCV isolated from PBS-treated cells, most of which appeared to encapsulate *Salmonella*. Without hepcidin treatment, FPN was detected by western blotting in the supernatant containing cellular debris and in the SCV fraction (Fig. 1c). When cells were pretreated with hepcidin, FPN was not detected in either fraction. Rab5 and MHC I, which are associated with SCV, as well as with the plasma membrane^10–12^, were detected in both fractions regardless of hepcidin treatment. Plasma membrane-bound annexin V^13^ was observed only in the supernatant fraction. These findings suggested that FPN is localized at or close to the SCV. To compare the effect of hepcidin before and after infection, Raw264.7 cells were infected with *Salmonella* for 1 h and then treated with hepcidin for an additional 1 h. The fractions obtained from the samples post-treated with hepcidin showed FPN in the SCV but not in the cell membrane fraction contrasting the data obtained in hepcidin pretreated samples. Of note, hepcidin could not cross the plasma membrane to act on FPN in the SCV (Fig. 1d).

**Fig. 2:**
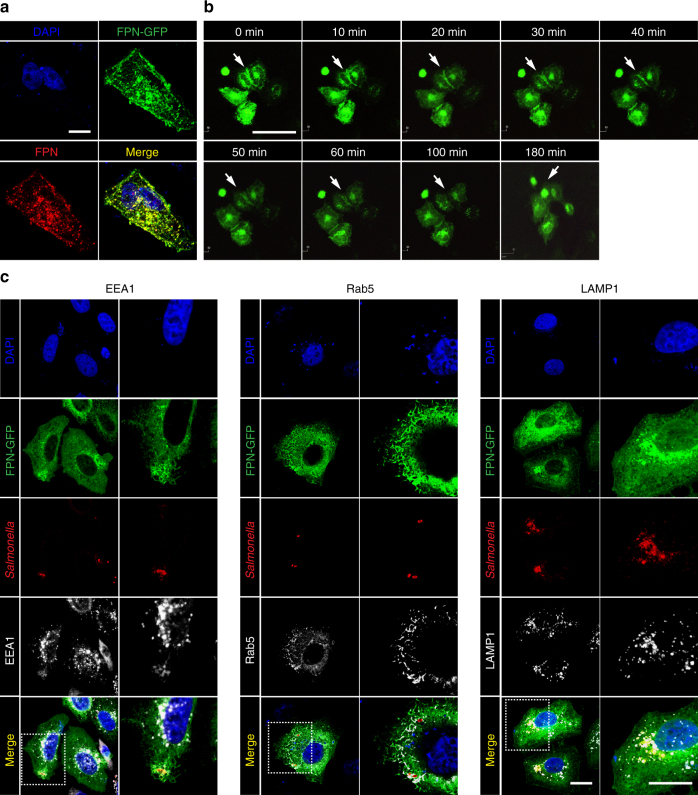
Localization of FPN with respect to SCV-associated marker proteins. **a** HeLa cells transfected with pFPN-EGFP were stained with an anti-FPN antibody and visualized by confocal microscopy. Scale bar, 10 μm. **b** Live cell images after treatment of HeLa cells expressing FPN-GFP with hepcidin. Arrows indicate FPN on the plasma membrane. Scale bar, 50 μm. **c** HeLa cells expressing FPN-GFP were infected with *Salmonella* (MOI, 100) and stained for the markers of early and late SCV (EEA1 and Rab5 at 1 h p.i. and LAMP1 at 12 h p.i.). Scale bar 10 μm. Images are representatives of over 100 infected cells from at least six independent experiments. The images on the left were taken at ×1000 magnification and on the right at ×2000 magnification, which were marked in dotted box on the left panel

SCV undergo modifications directed by proteins encoded by *Salmonella* Pathogenicity Islands (SPIs). Initially, the SCV associates with markers of early endosomes, such as early embryonic antigen (EEA)1, the transferrin receptor, and Rab5. This is later followed by acquisition of other lysosomal markers, such as lysosome-associated membrane protein (LAMP)1. Thus, we attempted to localize FPN during SCV maturation with these markers using HeLa cells expressing a FPN-GFP fusion protein. HeLa cells were transfected with the *fpn*-*gfp* fusion gene construct, and GFP expression was compared with that probed by specific anti-FPN antibody by confocal microscopy. Images of FPN-GFP showed a similar pattern of fluorescence as the images obtained with an anti-FPN antibody, suggesting that the GFP signal represented subcellular FPN localization (Fig. 2a). HeLa cells expressing FPN-GFP were next treated with hepcidin, and the GFP signal was evaluated by live cell imaging (Fig. 2b). Notably, the GFP signal at the cell membrane decreased in a time-dependent manner, and was almost completely abolished by 100–180 min. A GFP signal in the cytosol would represent FPN being processed through the endoplasmic reticulum and the golgi apparatus for transportation to the plasma membrane. We therefore evaluated the co-localization of FPN with EEA1 and Rab5, early SCV markers, and LAMP1, a later SCV marker. FPN-GFP localized immediately adjacent to EEA1 and Rab5 at 1 h p.i. and with LAMP1 at 12 h p.i., as observed by confocal microscopy (Fig. 2c). These were in marked contrast to the images of *Salmonella*-infected HeLa cells expressing GFP or uninfected HeLa cells expressing FPN-GFP (Supplementary Fig. 4), where GFP signals were not found near the SCV-associated markers. Therefore, it appeared that FPN is associated with the SCV at all stages of maturation.

### FPN acts as an iron transporter in the SCV membrane

**Fig. 3:**
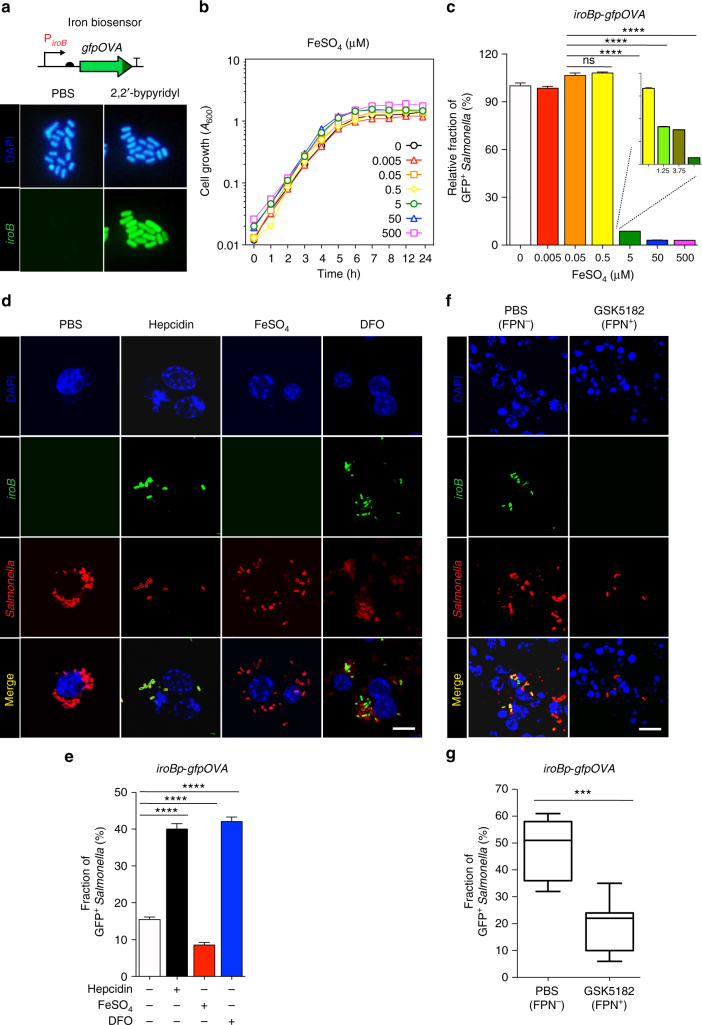
Assessment of SCV iron content in the presence or absence of FPN. **a** Expression of the iron biosensor in the presence or absence of iron chelator (2,2′-bypyridyl, 0.2 mM) in vitro. All *Salmonella* were stained with DAPI (blue), and *iroBp-gfpOVA*-expressing *Salmonella* are green. **b** *Salmonella* growth was determined in RPMI 1640 supplemented with the indicated concentration of FeSO_4_ (0.005–500 μM). Bacterial cell mass (*A*_600_) was measured at the indicated times. **c** Expression of *iroBp-gfpOVA* at 2 h p.i. in *Salmonella* grown as in **b** was quantified as the percentage of GFP^+^ *Salmonella*, as shown in **a**. Data are presented as means ± SEM. Significance is indicated as ****p < 0.0001 by two-tailed Student’s *t*-test. **d**, **f** Nuclei were stained with DAPI (blue), *Salmonella* with a specific antibody (red), and *iroBp-gfpOVA* expression is shown in green. **d** Expression of *iroB-gfpOVA* was determined in the *Salmonella* (MOI, 100) infecting Raw264.7 cells pretreated with PBS, hepcidin (1 μg/ml), FeSO_4_ (0.1 mM), or DFO (100 μM). Images were taken 2 h p.i. by confocal microscopy. **f** Expression of *iroB-gfpOVA* in the *Salmonella* infecting C57BL/6 mice treated with GSK5182 (FPN^**+**^) or PBS (FPN^**-**^). Representative images of spleens infected with *Salmonella* were examined by confocal microscopy 2.5 days after i.v. infection (1 × 10^5^ CFU). Scale bars 10 μm. **e**, **g** The fraction of *iroBp-gfpOVA*-expressing (green) *Salmonella* shown in **d**, **f** was quantified, respectively, as in **b**. Data are presented as means ± SEM. Significance is indicated as ***p = 0.0004; ****p < 0.0001 by two-tailed Student’s *t*-test

We then investigated the role of FPN on the physiology of *Salmonella* within SCV with respect to iron availability. As direct measurement of iron content in the SCV is virtually impossible, a biosensor was utilized. The tandem *iroN iroBCDE* gene cluster on the *Salmonella* chromosome is involved in the uptake of catecholate-type siderophore compounds^14^. These genes are highly induced under iron-limited growth conditions and repressed under iron sufficiency by binding of the Ferric Uptake Regulator (Fur)-Fe^2+^ repressor complex to operator sites in the cognate promoter region (data not shown). The expression of these genes, therefore, corresponds to changes in the number of iron atoms per bacterial cell. We took advantage of this iron responsiveness to assess iron conditions within the SCV. First, the iron responsiveness of the *iroB* promoter (*iroBp*) was verified using *Salmonella* carrying a reporter plasmid (*iroBp* fused to *lacZ*) (Supplementary Fig. 5A). To measure the activity of this promoter in intra-macrophage *Salmonella*, another gene reporter system was constructed using the unstable GFP variant gfpOVA^15^. The *gfp*OVA was cloned downstream of *iroBp* on a pSC101-based plasmid^16^ that was introduced into *Salmonella*. *Salmonella* expressing *gfp*OVA were used to monitor *iroBp* activity with or without an iron chelator by determining the fraction of GFP^+^ *Salmonella* (Fig. 3a). Quantification of the proportion of GFP^+^ *Salmonella* revealed that iron chelation increased *iroBp* activity about 20-fold (Supplementary Fig. 5B). The expression of the *iroB* gene transcript was quantified by qPCR and was also found to increase ~20-fold (Supplementary Fig. 5C). It is noteworthy that the expression of *iroB* was unaltered in response to other environmental changes such as magnesium concentration or pH shift (Supplementary Fig. 6). Taken together, these data demonstrate that *iroBp* is iron-responsive and that the fluorescence of *Salmonella* carrying *iroBp-gfpOVA* accurately reflected the *iroB* mRNA expression in an iron level-dependent manner. To determine the critical iron concentration for the modulation of *iroB* expression, *Salmonella* were grown in RPMI 1640, a chemically defined medium containing undetectable amounts of iron, supplemented with defined concentrations of FeSO_4_ (0.005–500 μM), and the *iroBp* activity was measured (Fig. 3b, c). Of note, *Salmonella* growth in the presence of increasing amounts of FeSO_4_ was indistinguishable, as reported previously (Fig. 3b)^17^. Fluorescence from *iroBp-gfpOVA* was repressed at iron concentrations between 0.5 and 5 μM in a concentration-dependent manner (Fig. 3c). The level of *iroB* mRNA determined by qPCR showed the same results (Supplementary Fig. 7). These data demonstrate that the *iroBp-gfpOVA* construct functioned accurately as an iron biosensor and that *Salmonella* could grow in the presence of minute (nM) concentrations of iron.

Hepcidin-induced degradation of FPN on the macrophage plasma membrane resulted in an ~30% increase in total cytosolic iron content (Supplementary Fig. 8). Changes in the iron level in the SCV in the presence or absence of FPN were assessed using *Salmonella* carrying the *iroBp-gfpOVA* biosensor. Raw264.7 cells cultured under iron overload conditions (0.1 mM FeSO_4_) or iron-depleted conditions 100 μM of the iron chelator deferoxamin (DFO)^18^ were infected with *Salmonella* (MOI, 100), and *iroBp* activity was determined at 1 h p.i. (Fig. 3d, e). The activity of *iroBp*, as determined by measuring the fraction of GFP^+^ *Salmonella*, increased in iron-depleted cells and decreased in iron-overloaded cells. Similar results were obtained when *iroB* mRNA levels were measured by qPCR (Supplementary Fig. 9). Most interestingly, depletion of FPN with hepcidin increased *iroBp* activity to the level observed in DFO-treated cells, suggesting that degradation of FPN reduced iron levels within the SCV in Raw264.7 cells. Upregulation of *iroB* with hepcidin was time-dependent, peaking at 2 h p.i. (Supplementary Fig. 10A). This indicates that FPN on the SCV acted as an iron transporter to move iron from the cytosol to the SCV, and depletion of FPN therefore resulted in iron-deficient conditions, leading to the activation of *iroBp*.

Finally, mice were infected with *Salmonella* carrying the *iroBp-gfpOVA* biosensor and treated with GSK5182, which abrogates hepcidin expression and consequently allows FPN to be maintained. The *iroBp* activity of the *Salmonella* in the splenic macrophages was determined 2.5 days p.i. (Fig. 3f, g). Quantification of GFP^+^ *Salmonella* (Fig. 3f) revealed that GSK5182 treatment repressed *iroBp-gfpOVA* expression by more than twofold (Fig. 3g). Downregulation of *iroBp* activity by GSK5182 treatment was confirmed by confocal microscopy, by counting the numbers of *Salmonella* (red) expressing GFP (green). Taken together, these data demonstrate that FPN on the SCV allows sufficient flow of iron into the SCV, resulting in downregulation of *iroBp* activity. By contrast, in the absence of FPN, the iron concentration in the SCV is reduced and *iroBp* activity is increased. FPN therefore acts as an iron transporter in the SCV membrane.

### Decreased iron in the SCV impairs ROS-dependent killing

**Fig. 4:**
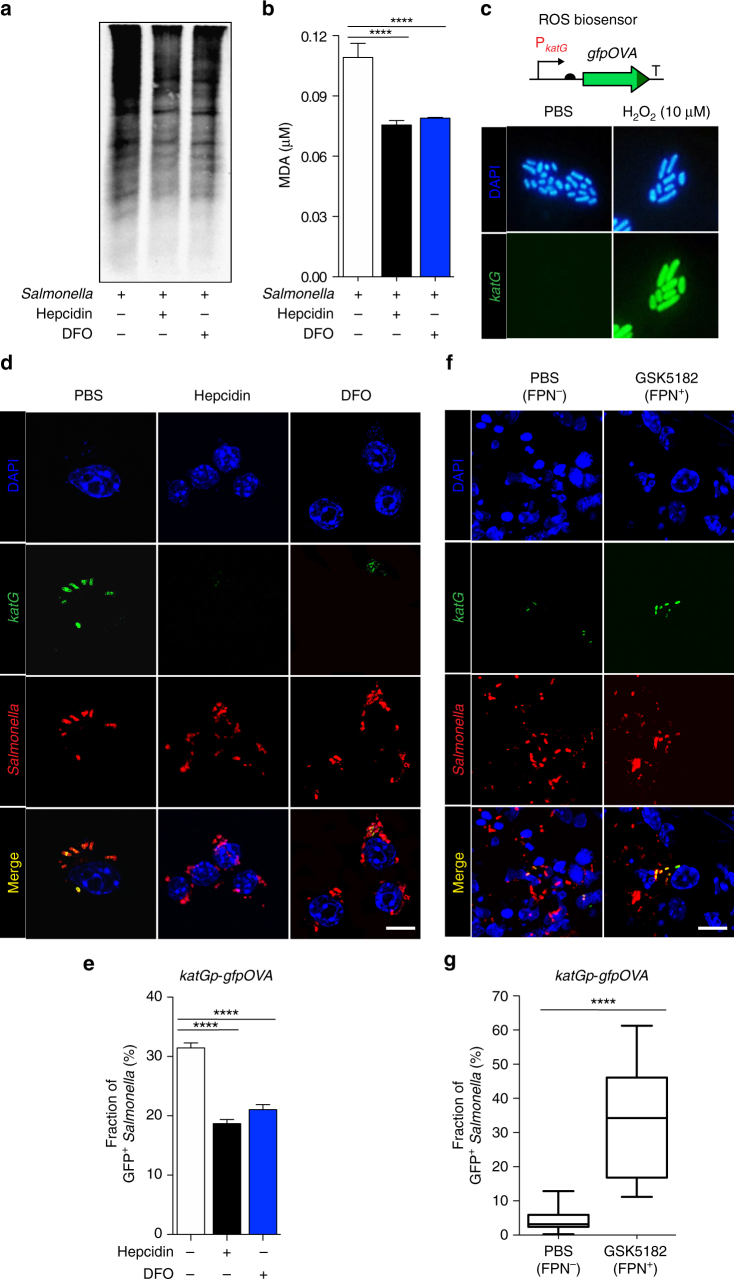
Assessment of ROS generation in SCV in the presence or absence of FPN. **a** Protein carbonylation in SCV of Raw264.7 cells treated with PBS, hepcidin, or DFO, as determined using 2,4-dinitrophenylhydrazine. SCV were isolated using magnetized *Salmonella* (MOI, 100) 2 h p.i. Proteins with 2,4-dinitrophenylhydrazine groups were quantified using a specific antibody. **b** Lipid peroxidation was determined in cells treated as in **a**. **c**–**g** ROS biosensor *Salmonella* (*katGp-gfpOVA*^16^) that fluoresce green in the presence of hydrogen peroxide were used to assess ROS levels in SCV. **c** Green fluorescence of *katGp-gfpOVA*-expressing *Salmonella* treated with 10 μM H_2_O_2_. **d** Expression of *katGp-gfpOVA* in the *Salmonella* infecting Raw264.7 cells pretreated with PBS, hepcidin (1 μg/ml), or DFO (100 μM). Images were taken 2 h p.i. using a confocal microscope. **f** Expression of *katGp-gfpOVA* in the *Salmonella* infecting C57BL/6 mice treated with GSK5182 (FPN^**+**^) or PBS (FPN^−^). Spleens of mice infected with *Salmonella* i.v. (1 × 10^5^ CFU) were examined by confocal microscopy 2.5 days p.i. **d**, **f** Nuclei were stained with DAPI (blue), *Salmonella* with a specific antibody (red), and *katGp-gfpOVA* expression is shown in green. Scale bars, 10 μm. **e**, **g** The fractions of biosensor-positive (green) cells were quantified as a percentage of the total intra-macrophage *Salmonella* (red) in the experiment shown in **d**, **f**. Data are presented as means ± SEM. Significance is indicated as ****p < 0.0001 by two-tailed Student’s *t*-test

In the early stages of *Salmonella* infection, there is an abrupt increase in superoxide formation known as the oxidative burst, which is catalyzed by a NADPH oxidase enzyme complex, a prototypical hemoprotein complex with heme iron at the active site^19–22^. The superoxide is then converted to hydrogen peroxide, which is further converted to other highly reactive hydroxyl radicals by the iron-catalyzed Fenton reaction^23,24^. Since downregulation of hepcidin by GSK5182 in mice infected with *Salmonella* conferred on antimicrobial effect^5^, we investigated whether the hepcidin-FPN axis is involved in ROS generation in macrophages. Hallmarks of ROS-mediated cellular damage include protein carbonylation^25^ and lipid peroxidation^26^. These parameters were therefore assessed as means of evaluating ROS generation in the SCV. Raw264.7 cells pretreated with hepcidin or with DFO, which prevents ROS generation by inhibiting the Fenton reaction^27^, were infected with magnetized *Salmonella*, and SCV were isolated 1 h p.i. SCV proteins were separated by SDS-PAGE and probed for carbonylated moieties using specific antibodies (Fig. 4a). There was an ~20% reduction in carbonylated proteins in SCV fraction from hepcidin-treated cells and an approximately 35% reduction in DFO-treated cells. Levels of malondialdehyde (MDA), the most abundant aldehyde produced by lipid peroxidation, were also measured by thiobarbituric acid-reactive substances (TBARS) assay (Fig. 4b)^26^. Hepcidin and DFO reduced MDA levels similarly. These results suggest that hepcidin treatment inhibited ROS generation.

To further verify the effect of hepcidin on ROS generation, *Salmonella* carrying an episomal *katGp*-*gfpOVA* fusion were used as a ROS biosensor^16^. The *katG* promoter is activated by the transcription factor OxyR upon exposure to H_2_O_2_ in a concentration-dependent manner (Fig. 4c)^16,28^. Raw264.7 cells pretreated with DFO or hepcidin were subsequently infected, and *katGp* activity was evaluated at 1 h p.i. The *katGp* activity in macrophages was determined by quantifying the fraction of GFP^+^ *Salmonella* (Fig. 4d, e). Under DFO treatment, which results in low ROS generation, the fraction of GFP^+^ bacteria was lower than in macrophages treated with PBS. Of note, treatment with hepcidin, which reduces FPN on the SCV, as well as on the plasma membrane, also reduced *katGp* activity. Similar results were observed when *katG* mRNA was measured by qPCR (Supplementary Fig. 11). The repressive effect of hepcidin on *katGp* activity was time-dependent (Supplementary Fig. 10B). Next, mice were infected with *Salmonella* expressing *katGp*-*gfpOVA* and treated with GSK5182, and the fraction of bacteria in the spleen expressing GFP was determined 2.5 days p.i. Confocal microscopy further revealed that GSK5182 treatment, which allows the maintenance of FPN, resulted in an ~10-fold increase in GFP^+^ *Salmonella* (Fig. 4f, g). ROS generation by NADPH oxidase and the iron-catalyzed Fenton reaction is therefore modulated by hepcidin, which presumably limits the availability of the iron cofactor in the SCV by modulating the maintenance of FPN.

**Fig. 5:**
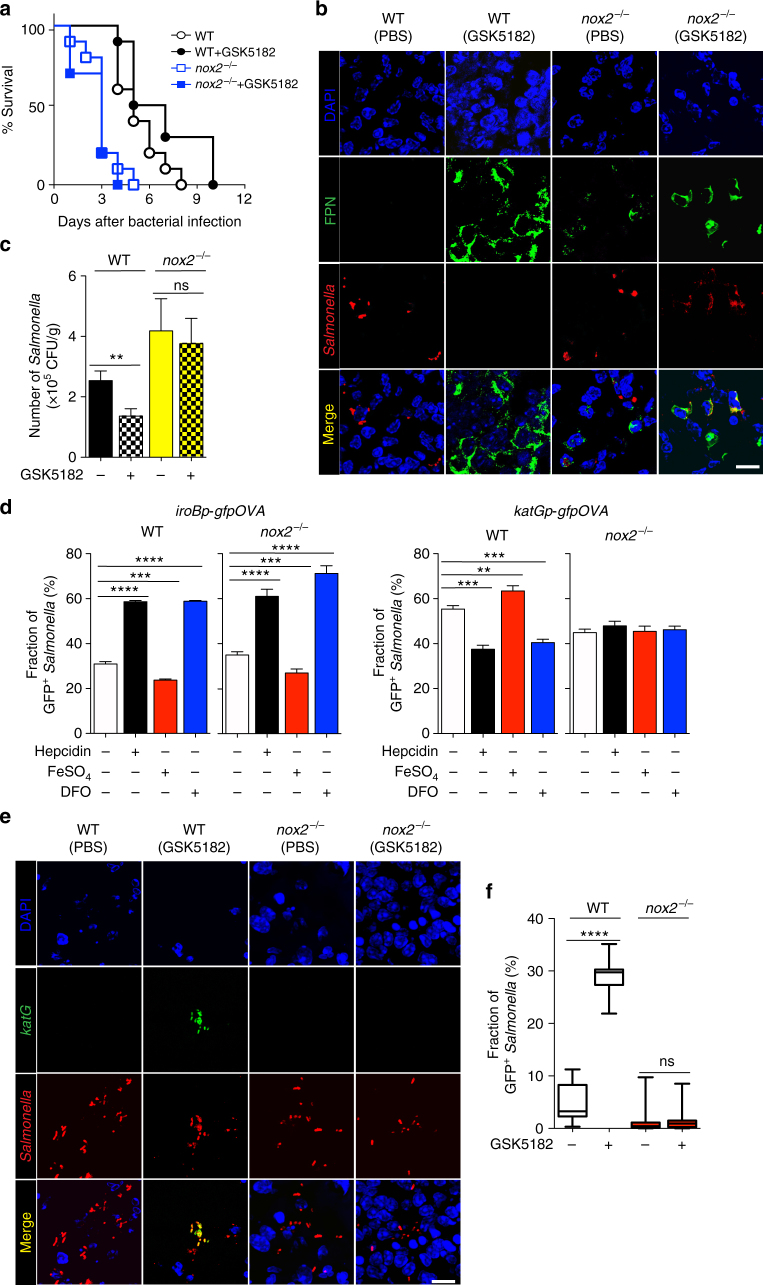
Effect of *nox2* mutation on *Salmonella* infection. **a** The survival of WT or *nox2*^*−/−*^ mice after oral infection with *Salmonella* (5 × 10^8^) with or without GSK5182 treatment (*n* = 10). According to log-rank, Mantel-Cox survival test, only WT mice showed overall survival gain with GSK5182 treatment upon *Salmonella* infection (*p* < 0.002). **b** WT and *nox2*^*−/−*^ mice treated with PBS (FPN^−^) or GSK5182 (FPN^+^) were infected with *Salmonella* (5 × 10^8^ CFU) orally. FPN and *Salmonella* in the spleen were examined by confocal microscopy 1.5 days p.i. using specific antibodies. Nuclei were stained with DAPI (blue), and *Salmonella* (red) and FPN (green) with specific antibodies. **c** Bacterial numbers (CFU/g) in the spleens of WT and *nox2*^*−/−*^ mice treated with GSK5182 were counted using the plating method. **d** Peritoneal macrophages were isolated from WT and *nox2*^*−/−*^ mice, treated with PBS, hepcidin (1 μg/ml), FeSO_4_ (0.1 mM), or DFO (100 μM), and infected with *Salmonella* carrying episomal *iroBp-gfpOVA* or *katGp-gfpOVA* biosensors. The fraction of intra-macrophage *Salmonella* expressing *gfpOVA* was determined. **e** WT and *nox2*^*−/−*^ mice were infected orally with *Salmonella* carrying the episomal *katGp-gfpOVA* biosensor (5 × 10^8^) and treated with PBS (FPN^+^) or GSK51812 (FPN^−^). Spleens were examined by confocal microscopy 1.5 days p.i. *Salmonella* are shown in red and *katGp-gfpOVA* expression is shown in green. **f** The fraction of *Salmonella* expressing *katGp-gfpOVA* in the experiment shown in **e**. Data are presented as means ± SEM. Significance is indicated as ***p < 0.001; ****p < 0.0001 by two-tailed Student’s *t*-test

This model predicts that GSK5182 would not be effective in controlling *Salmonella* infection in mice deficient in *nox2*, which lack the gp91 component of NADPH oxidase, the main cellular source of ROS^29^. To test this hypothesis, wild-type (WT) and *nox2*^*−/−*^ mice were orally infected with *Salmonella* (1 × 10^8^) and treated with GSK5182 or PBS, and the survival of infected animals was determined. In contrast to WT mice, GSK5182 did not have any therapeutic effect in the *nox2*^*−/−*^ mice; in fact, *nox2*^*−/−*^ mice expired earlier than WT mice (Fig. 5a). The spleens of these mice were examined for FPN expression 2.5 days p.i. (Fig. 5b and Supplementary Fig. 12). FPN was expressed in the spleens of both WT and *nox2*^*−/−*^ mice treated with GSK5182. Apparently, GSK5182 abrogated hepcidin activity and allowed FPN to be maintained in splenic macrophages^5^. The bacterial loads of *Salmonella* in the spleen were determined by counting colony forming units (Fig. 5c). Consistent with the survival data (Fig. 5a), the bacterial load in WT mice was about half of that in the *nox2*^*−/−*^ mice. However, although GSK5182 reduced *Salmonella* numbers in WT mice, GSK5182 had little effect on the bacterial load in *nox2*^*−/−*^ mice. Subsequently, peritoneal macrophages from WT and *nox2*^*−/−*^ mice were isolated, pretreated with DFO, FeSO_4_, or hepcidin, and infected with *Salmonella* carrying the episomal *iroBp-gfpOVA* or *katGp-gfpOVA* biosensors (Fig. 5d). The *iroBp* activity, which was determined using the fraction of GFP-expressing bacteria as a surrogate, increased in macrophages treated with hepcidin (FPN^−^; high-iron level) and macrophages treated with DFO (FPN^+^; low-iron level), and decreased in macrophages pretreated with FeSO_4_ (FPN^+^; high-iron level). This was the case in macrophages derived from both WT and *nox2*^*−/−*^ mice. By contrast, *katGp* activity decreased in WT macrophages treated with hepcidin or DFO and increased in WT macrophages treated with FeSO_4_. Most interestingly, however, *katGp* activity in the *nox2*^*−/−*^ macrophages did not change in response to hepcidin, DFO, or FeSO_4_, suggesting that hepcidin affected *nox2*-derived ROS. This finding was further verified using *nox2*^*−/−*^ mice. The effect of GSK5182 was tested in WT and *nox2*^*−/−*^ mice infected with *Salmonella* expressing *katGp-gfpOVA*. The activity of *katGp* in the spleen was determined by quantifying the fraction of bacteria expressing GFP^+^ *Salmonella* at 1.5 day p.i. (Fig. 5e, f). In the absence of GSK5182, 71% of *Salmonella* expressed GFP in WT mice, while only 15% of *Salmonella* in *nox2*^*−/−*^ mice were GFP^+^. GSK5182 treatment further enhanced *katGp* activity in WT mice but not in *nox2*^*−/−*^ mice. Taken together, these findings indicate that the anti-*Salmonella* effect of GSK5182 treatment is dependent on ROS generation, which is affected by the presence or absence of FPN in the SCV.

**Fig. 6:**
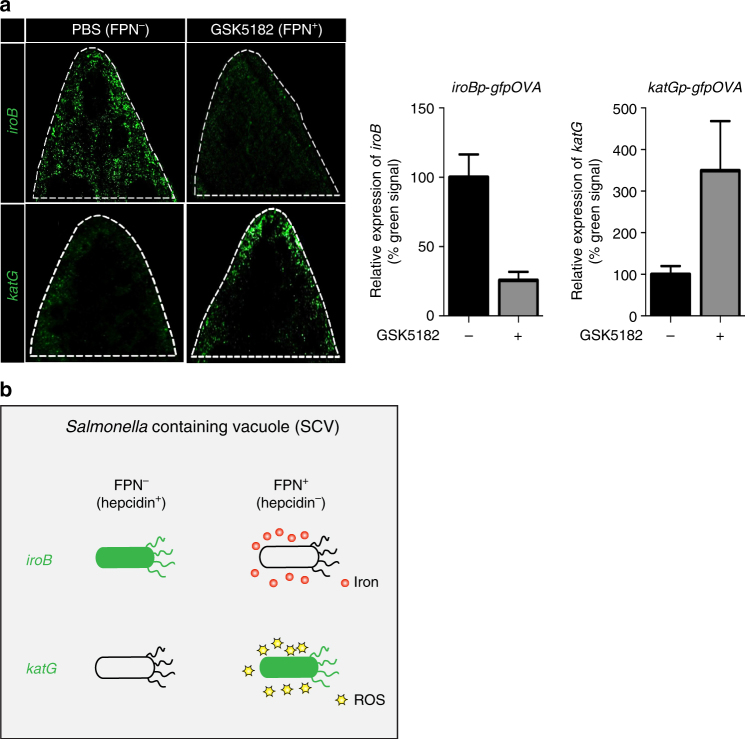
Inverse correlation between iron level and ROS generation. **a** WT mice were infected with *Salmonella* carrying *iroBp-gfpOVA* or *katGp-gfpOVA* plasmid and treated with GSK5182 or PBS. Whole spleens were isolated 2.5 days p.i. and examined for *iroB* or *katG* expression by fluorescence microscopy. The relative expression of *iroB* or *katG* in the infected spleen shown in Supplementary Fig. 13 was determined by calculating relative intensity of green signal (% = intensity of green signal from GSK5182 treated/intensity of green signal from PBS treated) using ImageJ 1.41 (NIH). Significance for *iroB* is *p* = 0.0091; for *katG*, *p* = 0.0238 by two-tailed Student’s *t*-test. **b** Graphical summary

Finally, WT mice were infected with *Salmonella* carrying *iroBp-gfpOVA* or *katGp-gfpOVA* and treated with GSK5182 or PBS. Whole spleens were isolated 2.5 days p.i. and examined for *iroB* or *katG* expression by fluorescence microscopy (Fig. 6a and Supplementary Fig. 13). In PBS-treated mice, in which FPN is degraded, *iroB* was expressed but *katG* was not. In GSK5182-treated mice, in which FPN levels are maintained, *iroB* expression decreased while *katG* expression increased. These results show that the maintenance of FPN leads to iron-sufficient conditions in the SCV for ROS generation (Fig. 6b).

## Discussion

The initial stages of SCV formation and the composition of the SCV are only poorly understood. The evidence presented in this study suggests that FPN is selectively internalized along with *Salmonella* in macrophages, and transports iron into the SCV lumen as supported by the functional analyses (Figs. 1 and 3). FPN1 protein was also localized in *M*. *tuberculosis* phagosomes after infection of RAW264.7 macrophages^30^. In this case, however, the FPN1 was suggested to be involved in the export of iron from the phagosome into the cytosol. The *Salmonella* iron biosensor (*iroBp*-*gfpOVA*) was activated at below μM concentrations of iron and was repressed at higher levels. This suggests that iron levels in the SCV fluctuate in the μM range. It must be emphasized here that *Salmonella* grows normally when iron levels are in the nM range, presumably by producing the catecholate siderophores enterobactin and salmochelin^31^. The iron released from the hydrolyzed siderophores would then allow the survival and proliferation of intra-macrophage *Salmonella*. Thus, the concept of ‘nutritional immunity’ in the control of *Salmonella* must be used with caution, as *Salmonella* only require a minute quantity of iron for growth and also reside in a separate compartment within the macrophage. Thus, it is the presence or absence of FPN on the SCV membrane that determines iron levels in the SCV lumen: depletion of FPN increased cytosolic iron content but decreased the iron level in the SCV (Fig. 3 and Supplementary Fig. 8).

Generation of ROS is initiated by heme iron of flavocytochrome b558 in NADPH oxidase transferring electrons from NADPH to oxygen which is reduced to superoxide anion^23,24,32^. It is inferred that this activity of the NADPH oxidase must be functionally correlated with the availability of iron for insertion of heme prosthetic group into the apoprotein. Our results revealed that the iron level in SCV influenced ROS generation rather than *Salmonella* proliferation: *Salmonella* survived better in iron-deficient conditions (no FPN) than in iron-sufficient conditions (with FPN) in vivo (Fig. 5). Studies with ROS biosensors revealed that hepcidin, which induces FPN degradation, reduced ROS generation in the SCV, as determined by measurement of oxidation of macromolecules, as well as *katGp* activity both in vitro and in a murine model (Fig. 4). A similar effect was reported in mice treated with DFO, which increased intracellular replication of *Salmonella*^27^. However, iron chelation has inhibitory effects on the replication of intra-macrophage *Salmonella* in iron starvation models based on another iron chelator, deferasirox^33–35^. We propose here that reduction of the available iron specifically in the SCV attenuates the production of hydroxyl radicals, which accounts for the capacity of these cells to limit intra-macrophage *Salmonella* growth^36^ (Supplementary Fig. 14). This model was based on the observation that depletion of FPN with hepcidin reduced iron intake into the SCV, which is needed for generation of antibacterial ROS in the SCV by NADPH oxidase and Fenton reaction (Fig. 4). Consequently, the increased survival of *Salmonella*-infected wild-type mice treated with GSK5182 was modest (Fig. 5a), possibly because ROS confer a critical antimicrobial effect early during the respiratory burst as *Salmonella* infect host cells anew^37^—GSK5182 removes only a part of various antimicrobial defenses in macrophages. It should be noted that iron-dependent ROS generation in SCV under this experimental condition was only ~20% (Fig. 4a, b), which might be sufficient to damage *Salmonella* without harming the organelles of the host cell. A similar ROS-dependent mechanism could account for the control of infection by intracellular bacteria such as *Legionella pneumophila*, and *Mycobacterium tuberculosis* and *avium*, known to reside in membrane-bound cytoplasmic vacuoles in macrophages^38–40^. In addition, the iron transporter natural resistance-associated macrophage protein 1 (Nramp1) was suggested to be involved in the regulation of iron level in SCV. Thus, mutations at *Nramp1* cause susceptibility to infections by several intracellular pathogens, including *Salmonella*^41–43^. Therefore, we have executed the same set of experiments with peritoneal macrophages isolated from DBA2 mice (Nramp1^*+/+*^) to clarify the function of Nramp1 in regards to iron level in SCV. We observed similar patterns of *iroB* and *katG* responses; *iroB* was increased and *katG* was decreased following hepcidin treatment (Supplementary Fig. 15). Consistently, we have previously observed and reported a similar protective effect of GSK5182 in DBA2 mice^5^. Taken together, this study provides a mechanism underlying the control of intracellular bacterial infection by ERRγ inverse agonist, GSK5182, although further studies would elucidate the role of Nramp1 in conjunction with FPN in intracellular bacterial infection.

## Methods

### Bacterial strains, plasmids and culture conditions

*Salmonella typhimurium* SL1344 was used as the WT strain. WT *Salmonella* and the p*katGp*-*gfpOVA* plasmid were described previously^16^. The *iroB* reporter plasmid (p*iroBp*-*gfpOVA*) was constructed by replacing the *katG* promoter of the p*katGp*-*gfpOVA* plasmid with the *iroB* promoter region (−200 to +100) using the Sph1 and Xba1 restriction enzymes. The pFPN-EGFP plasmid was generated by ligating Xba1 and EcoR1 fragments of full-length *fpn* and pEGFP-N1 (Clontech). Bacterial cultures were grown in Luria broth (LB; Difco Laboratories) containing 1% NaCl (w/v) with vigorous aeration at 37 °C, and, when necessary, ampicillin (Sigma, #A0166) was added at 100 μg/ml, unless otherwise indicated. For solid support medium, Bacto agar (Difco Laboratories) was included at 1.5% (w/v).

### Measurement of bacterial growth

Overnight bacterial cultures were diluted 1:100 into fresh LB and cultured for 3 h at 37 °C. The iron chelator 2,2′-bipyridyl (Sigma, #D216305) was added, and bacteria were further cultured to test the impact of iron deprivation on bacterial growth. To examine the effect of iron concentration on bacterial growth, RPMI 1640 (Gibco/Thermo Fisher Scientific, #1785905) was chosen as the culture medium. Overnight cultures were grown in RPMI 1640 medium containing FeSO_4_ (0.005–500 μM), and then diluted 1:100 into fresh medium containing the same concentration of iron and further incubated at 37 °C. Bacterial growth was measured every hour at an absorbance of 600 nm (*A*_600_).

### Culture of Raw264.7 macrophages, HeLa cells, and peritoneal macrophages

RAW264.7 and HeLa cell lines were obtained from ATCC Korea and cultured according to the supplier’s recommendation. Primary peritoneal macrophages were isolated from 8-week-old C57BL/6 J mice (Jackson Laboratory) 3 days after elicitation by injection of 3% thioglycollate into the peritoneal cavity (1 ml per mouse). The isolated macrophages were cultured in RPMI 1640 supplemented with 10% fetal bovine serum (FBS) in an incubator with 5% CO_2_^44^. The macrophages were seeded at 5 × 10^5^ cells per ml for all experiments.

### Localization of FPN in HeLa cells upon *Salmonella* infection

HeLa cells were transiently transfected with pFPN-EGFP using FuGENE HD transfection reagent (Promega, #E2311). Live cell images were taken of HeLa cells expressing FPN-GFP after treatment with hepcidin using the IncucyteS3 Live Cell Analysis System at ×20 magnification (Essen Biosciences, USA). HeLa cells expressing FPN-GFP were stained with the following antibodies to SCV-associated marker proteins up to 12 h after *Salmonella* infection (MOI, 100): anti-EEA1 (Abcam, #ab18211), anti-Rab5 (Abcam, #ab2900), and anti-LAMP1 (Abcam, #ab24170). Fluorescent signals were imaged with a LSM 880 confocal laser scanning microscopes equipped with VIS and NIR lasers. All captured images were taken using the Airyscan mode supported by the LSM 880 confocal laser scanning microscopy for image optimization (Carl Zeiss).

### Measurement of gene expression using biosensors

Fluorescence of bacteria expressing the p*iroBp*-*gfpOVA* was measured in vitro using SpectraFluor Plus (Tecan, Austria) at an excitation of 470 nm and an emission of 510 nm. To measure the expression of *iroB* or *katG* by *Salmonella* within macrophages, Raw264.7 cells or peritoneal macrophages were infected with *Salmonella* expressing the p*iroBp*-*gfpOVA* or p*katGp*-*gfpOVA*, respectively. Infected macrophages were fixed at 1 h p.i. with 4% paraformaldehyde for 10 min at 4 °C. The fixed cells were washed with PBS three times and then treated with ProLong Gold anti-fade reagent with 4′,6-diamidino-2-phenylindole (DAPI; Invitrogen/Thermo Fisher Scientific, #P36935). *Salmonella* were stained with an anti-*Salmonella* primary antibody (Abcam, #ab8274) and an AlexaFluor 568-conjugated goat anti-mouse secondary antibody (Invitrogen/Thermo Fisher Scientific, #A11031) (red). The stained cells were imaged on a fluorescence microscope and the expression of *iroB* or *katG* was quantified by comparison of the green and red signals in a single image using ImageJ 1.41 (NIH). A total of 1712 images were analyzed.

### Isolation of SCV

For isolation of SCV within Raw264.7 cells, magnetite particles (Bangs Laboratories, #BM570) were used. The particles were prepared as described previously^13^. Briefly, the particles were washed twice with 0.1 M MES buffer [2-(*N*-morpholino) ethanesulphonic acid, pH 5.2] using a magnetic rack after centrifugation of the stock suspension (1 ml) at 1000×*g* for 30 s in a fixed-angle centrifuge. The washed particles were then resuspended with 4 mg ml^−1^ EDAC [1-ethyl-3-(3-dimethylaminopropyl) carbodiimide], and mixed on a rotator (12 r.p.m.) for 15 min at RT. The activated particles were washed twice with PBS (pH 7.4) using magnetic separation. The prepared particles were mixed with 0.5 × 10^9^ live *Salmonella* and incubated at 37 °C on a rotator (12 r.p.m.) for 30 min. Raw264.7 cells were infected with the magnetized *Salmonella* at a MOI of 100. After infection, the cells were washed two times with DPBS (Well Gene, #LB001-02) containing phenylmethanesulfonyl fluoride (PMSF) (10 mM; Sigma, #P7626), and then treated with enzyme-free cell dissociation solution (Millipore, #S-004-B) in a 5% CO_2_ incubator at 37 °C for 2 min. To inactivate the dissociation solution, RPMI containing PMSF (10 mM) was added, and then the detached cells were transferred into a microfuge tube placed on a magnetic rack (Bioneer, MagListo-2, TM-1010). After washing with DPBS containing PMSF, the cells were fragmented with a Dounce homogenizer (Wheaton, #357542), and then with a Polytoron homogenizer (IKA, Ultra-Turrax T 10), and then sonicated (Sonics, Vibra Cell VC505/VC750). Finally, purified SCV were isolated using a magnetic rack. The SCV were analyzed by confocal microscopy or lysed by mixing with RIPA buffer (Bio-solution, #BR002) containing a cocktail of proteinase K and protein phosphatase inhibitors (Thermos, #RB228066, #RA229831) on ice for western blot analysis.

### Western blot analysis of FPN

Macrophages pretreated with hepcidin or PBS for 3 h were infected with *Salmonella*. The SCV were isolated from whole-cell lysates of macrophages as described above, and the remaining fraction (non-SCV fraction: supernatant) was analyzed by western blot. The samples (50 μg) were run on 10% SDS- polyacrylamide gels and transferred to PVDF membranes (Amersham, #10600002). Primary antibodies were diluted 1:3000 in TBST (TBS with 0.2% Tween-20) and incubated for 16 h at 4 °C. The following primary antibodies were used: anti-FPN (Novus Biologicals, #NBP1-21502), anti-Rab5 (Abcam, #ab18211), anti-annexin V (Abcam, #ab14196), and anti-MHC class I (Novus Biologicals, #NB120-15680). After washing with TBST three times, membranes were incubated with a 1:3000 dilution of horseradish peroxidase (HRP)-conjugated anti-rabbit (Thermo Fisher Scientific, #31430) or anti-rat (Thermo Fisher Scientific, #31470) secondary antibodies in TBST for 1 h at RT. After washing, the signals were visualized using chemiluminescence (Pierce, #32106) and the Fusion Solo (Vilaber) imaging system. Uncropped images of blots are shown in Supplementary Fig. 16.

### Analysis of ROS-related damage

The level of protein carbonylation was determined using the oxidized protein detection kit (Millipore, #S7150-Kit). The lysed SCV were separated by SDS-PAGE and blotted on PVDF membranes, followed by western blot analysis^45,46^. In addition, ROS-related lipid peroxidation was estimated by thiobarbituric acid (TBA)-MDA assay using the TBARS assay kit (R&D Systems, #KGE013). The amount of lipid peroxidation in the sample was quantified by measuring TBARS levels at 532 nm using a SpectraFluor Plus instlument^26^.

### Animal experiments

Male and female 8-week-old C57BL/6J WT and *nox2*^*−/−*^ mice (B6.129S-*Cybb*^*tm1Din*^/J, stock number: 002365) were from the Jackson Laboratory (Bar Harbor, USA). The hepcidin inhibitor GSK5182 was synthesized as described previously^5^. To investigate the effect of GSK5182, mice were injected with PBS or GSK5182 (40 mg kg^−1^) intraperitoneally 12 h before *Salmonella* infection (*n* = 5 per group). After bacterial infection, mice were injected with PBS or GSK5182 once daily. Mice were infected with 1 × 10^8^ CFU of *Salmonella* orally or with 1 × 10^5^ CFU of *Salmonella* i.v. To determine the viable counts of *Salmonella*, spleens from five mice per group were collected and homogenized in sterile PBS containing 0.05% Tween-20 with a Polytoron homogenizer (IKA, Ultra-Turrax T 10). *Salmonella* was recovered from the homogenates and quantified by plating on agar. For the survival assay, 20 C57BL/6J WT or *nox2*^*−/−*^ mice were infected orally with 1 × 10^8^ Salmonella with or without GSK5182 treatment (*n* = 10 per group). Survival rates were estimated by the Gehan-Breslow-Wilcoxon test. All animal experiments were approved and performed according to guidline of the Institutional Animal Use and Care Committee of the Chonnam National University (CNU IACUC-H-2016-15). For measurement of the expression of *katG* or *iroB*, mice were infected i.v. with 1 × 10^5^ CFU of *Salmonella* carrying the p*katGp*-*gfpOVA* or p*iroBp*-*gfpOVA* plasmid, respectively. Gene expression was evaluated by analysis of red and green fluorescence using ImageJ, as described above.

### Immunofluorescence staining and confocal microscopy

Raw264.7 cells were grown on sterilized glass cover slips for 12 h. Pretreatments and *Salmonella* infection were carried out as described above. The cells were then fixed with 4% paraformaldehyde and blocked with 3% bovine serum albumin (BSA) in PBS. The samples were probed with a mouse anti-*Salmonella* antibody (1:200 in PBS; Abcam, #ab8274) and an AlexaFluor 568-conjugated secondary antibody (1:500 in PBS; Invitrogen/Thermo Fisher Scientific). The nuclei of the macrophages were stained with ProLong Gold anti-fade reagent with DAPI (Invitrogen/Thermo Fisher Scientific, P36935).

For in vivo experiments, the isolated spleens were fixed with 4% paraformaldehyde, embedded in optimal cutting temperature compound (OCT; Tissue-Tek), and frozen. The frozen spleens were sliced into 6-μm-thick sections using a microtome-cryostat. The tissue sections were mounted on aminopropyltriethoxysilane-coated slides. The slides were washed with PBS (pH 7.4) for complete removal of the OCT and incubated with primary antibodies [rabbit anti-mouse FPN (Alpha Diagnostic, #MTP 11-A) and mouse anti-*Salmonella* antibody (Abcam, #ab8274)] at 1:200 in PBS for 2 days at 4 °C. AlexaFluor 568-conjugated goat anti-mouse and AlexaFluor 488-conjugated goat anti-rabbit were used as secondary antibodies (1:100 in PBS). The samples were also stained with ProLong Gold anti-fade reagent with DAPI to detect nuclei. The stained images were captured with a Zeiss confocal microscope LSM 510 (Zeiss Laboratories) and the representative images were shown unless otherwise mentioned.

### Statistical analysis

Data were analyzed using GraphPad Prism statistical software. The data met assumptions of a normal distribution as determined by statistical software, and variance was estimated using the standard error of the mean (SEM). The two-tailed Student’s *t*-test was used to analyze differences between two groups. Differences were considered statistically significant at *p* < 0.05.

### Data availability

The data supporting the findings of the study are available in this article and its Supplementary Information files, or from the corresponding author upon request.

## Electronic supplementary material

Supplementary Information
