# Supplementary material for: The hepcidin-ferroportin axis controls the iron content of Salmonella-containing vacuoles in macrophages
Source: Nat Commun. 2018 May 29;9:2091. doi: 10.1038/s41467-018-04446-8 (PMC5974375; doi:10.1038/s41467-018-04446-8)
Supplement: Supplementary file 1 — Supplementary Information [file 41467_2018_4446_MOESM1_ESM.pdf]

Hepcidin-ferroportin axis controls the iron content of *Salmonella*-containing vacuoles in macrophages

Lim D. *et al*

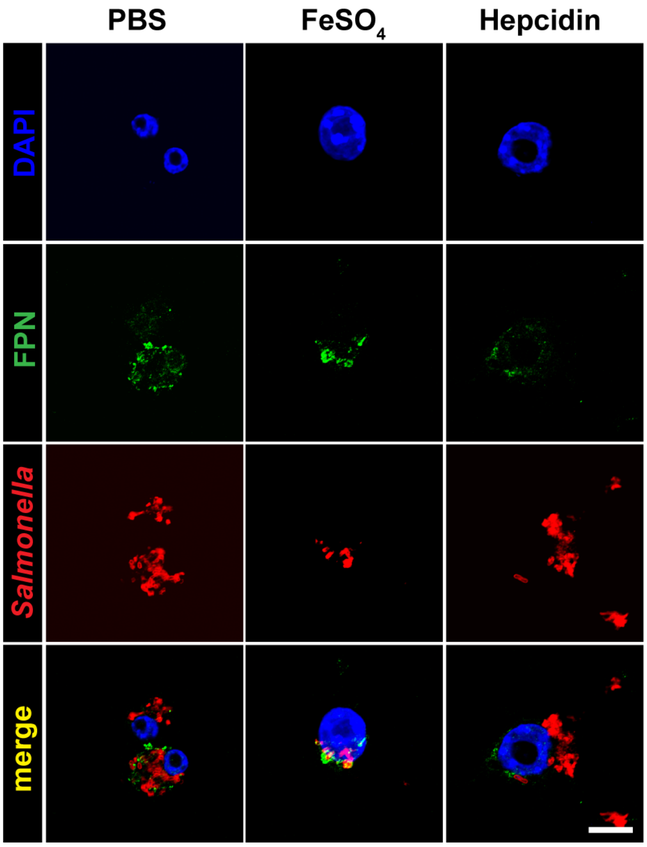

Supplementary Figure 1. Raw264.7 cells pretreated and infected with *Salmonella* for 12 hr were analyzed as described in Figure 1A.

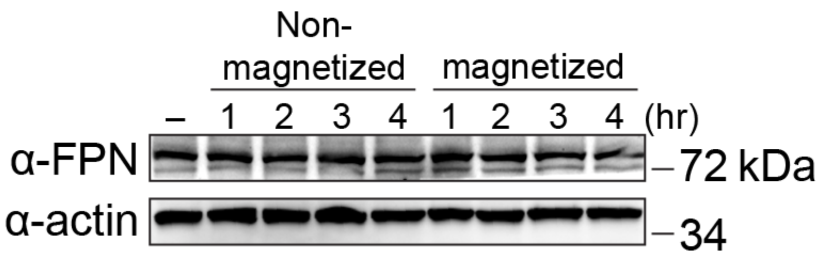

Supplementary Figure 2. Level of FPN in *Salmonella* infected Raw264.7 cells. The level of FPN from infected Raw264.7 cells was analyzed by Western blot analysis using anti-FPN antibody. Raw264.7 cells were infected either with non-magnetized or magnetized *Salmonella* and total protein was isolated at the indicated time after post-infection. Total protein from uninfected Raw264.7 cells was used as a control (lane 1; -).

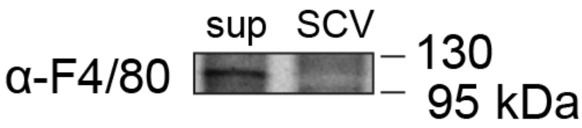

Supplementary Figure 3. Detection of a murine macrophage surface marker from plasma membrane. Representative murine surface glycoprotein, F4/80, was detected in the supernatant containing cellular debris (lane1; sup) but not in the SCV fraction (lane 2; SCV) as analyzed by Western blotting using anti-mouse F4/80 antibody (Bio-rad #MCA497GA).

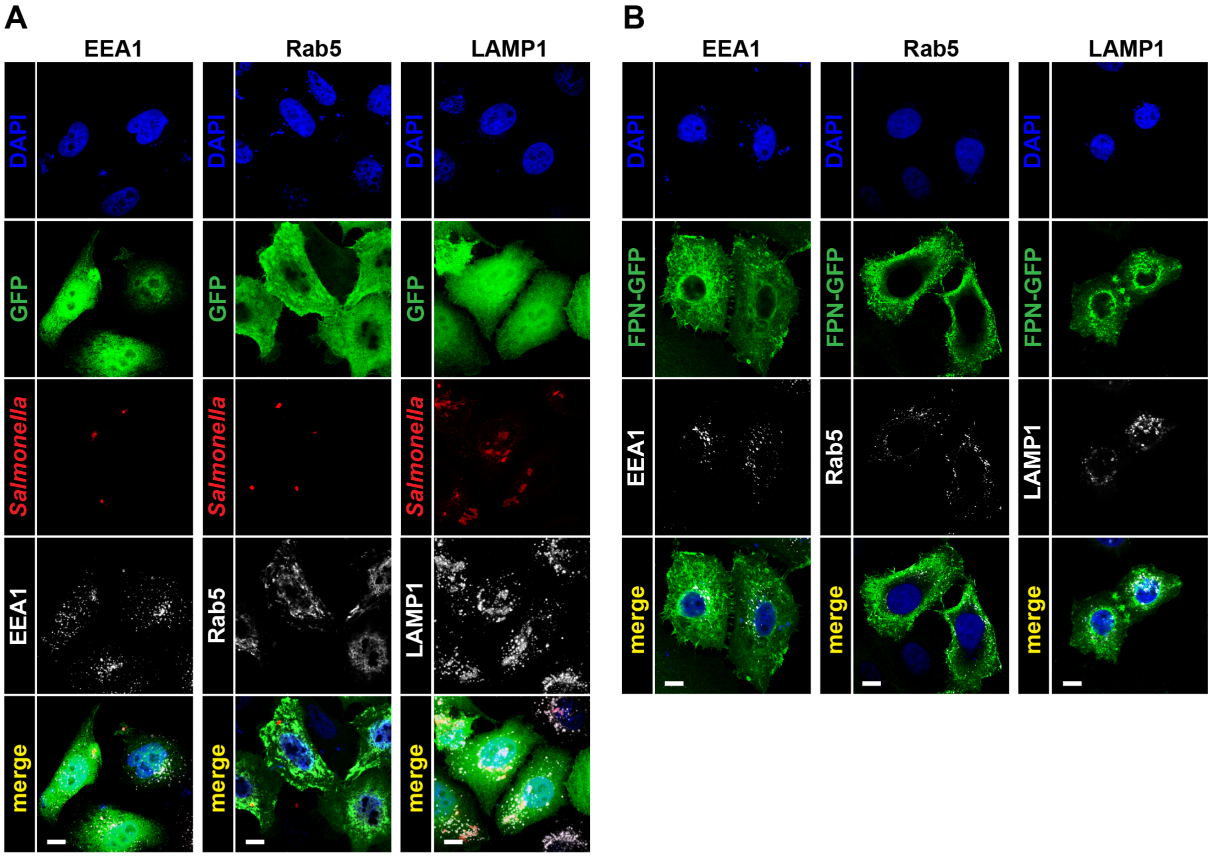

Supplementary Figure 4. Images of membrane markers from infected HeLa cells expressing GFP (mock, pEGFP-N1) (A) or uninfected HeLa cells expressing FPN-GFP (B) as negative controls for Figure 2. HeLa cells expressing GFP or FPN-GFP were stained for the markers of early and late SCV (EEA1 and Rab5 at 1 hr *p.i.* and LAMP1 at 12 hr *p.i.*). Scale bar, 10 μm. Images are representatives of over 100 cells from at least 3 independent experiments.

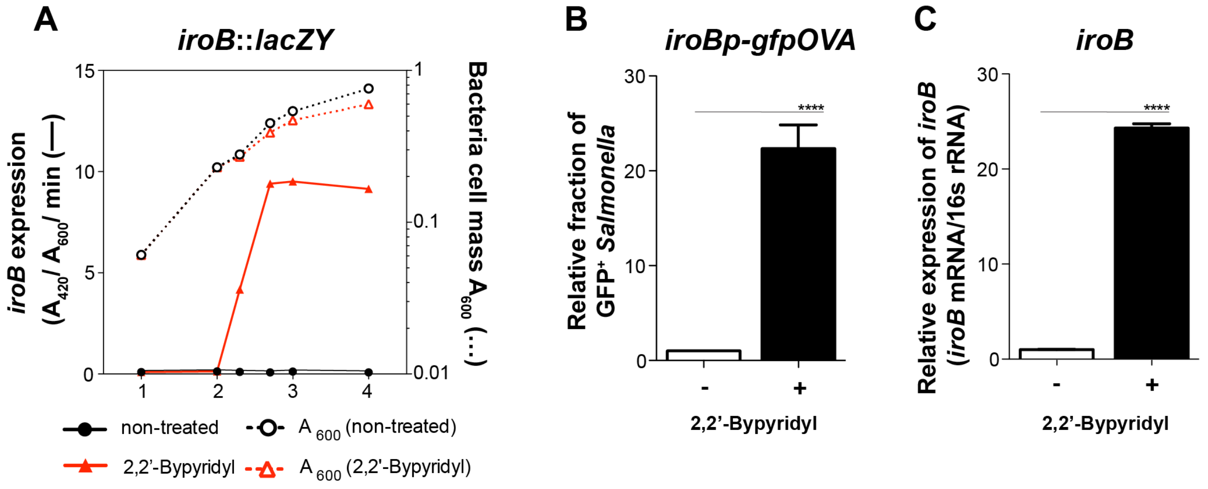

Supplementary Figure 5. Fur-dependent expression of *iroB* under iron-depleted conditions. (A) Expression of *iroBp* was induced during *Salmonella* growth in LB upon addition of an iron chelator (2,2'-bypyridyl, 0.2 mM). The *iroBp* activity was determined using episomal *iroBp* fused to the *lacZ* reporter gene. Bacterial cell mass (A_{600}) and differential β-galactosidase activity (A_{420}/A_{600}/min) were determined at the indicated times. (B, C) Activity of *iroBp* in *Salmonella* carrying the episomal *iroBp-gfpOVA* reporter grown in LB in the absence or presence of 2,2'-bypyridyl. Bacterial samples were measured at $A_{600} = 0.5$. The *iroBp* activity was determined by measuring the fluorescence of bacterial cultures with a fluorometer (B) or by measuring *iroB* mRNA relative to 16S rRNA by qPCR (C). Significance is indicated as ****$p \leq 0.0001$.

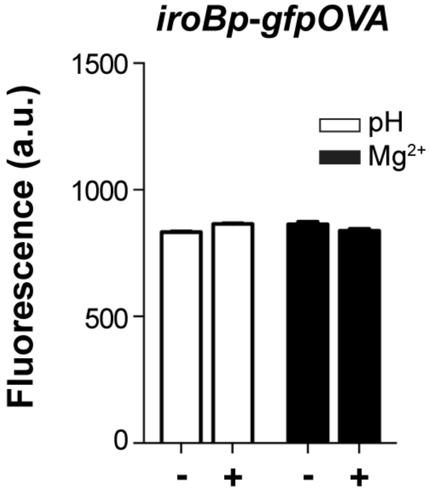

Supplementary Figure 6. Activity of *iroB*p under pH or Mg^{2+} concentration shift condition. Bacteria were cultured in RPMI media supplemented with 0.5 μM $FeSO_4$. When A_{600} reached to 0.2, the culture was washed with new media at pH7 (white bar, -) or pH4.4 (white bar, +) for pH shift and further incubated for 2 hr. For Mg^{2+} concentration shift sample, bacteria were grown in RPMI media containing 0.5 μM $FeSO_4$ and 10 mM $MgCl_2$. The culture was washed with fresh media containing 0.5 μM $FeSO_4$ and either 10 mM (black bar, -) or 8 μM $MgCl_2$ (black bar, +) at A_{600}=0.2 then grown for 2 hr. The expression level of *iroB*p-*gfp*OVA was measured as a fluorescence intensity (a.u.) using SpectraFluor Plus (Tecan, Austria) at an excitation of 470 nm and an emission of 510 nm.

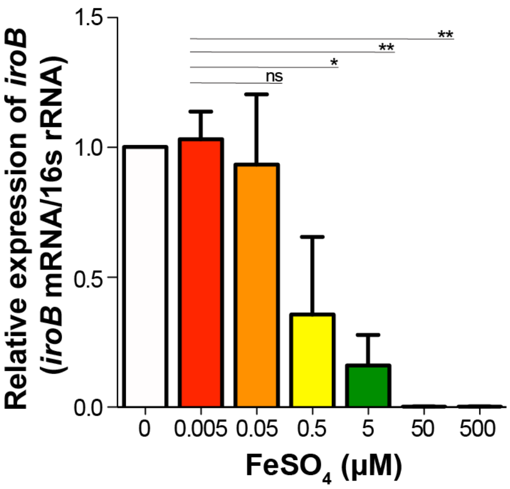

Supplementary Figure 7. Activity of *iroBp* in *Salmonella* grown in RPMI 1640 supplemented with the indicated concentrations of $FeSO_4$ (0.005–500 μM) by quantifying the ratio of *iroB* mRNA to 16S rRNA by qPCR. Significance is indicated as ns, not significant; *p=0.0114; **p=0.0039

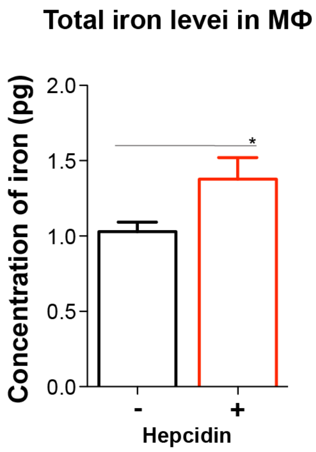

Supplementary Figure 8. Total iron content in *Salmonella*-infected Raw264.7 cells after hepcidin treatment. Raw264.7 cells (1 × 10^6/well) were pretreated with hepcidin (1 μg/ml, 3 hr), and then infected with *Salmonella* at a MOI of 100. Total intracellular iron was measured 1 hr *p.i.* using the Iron Assay kit (Abcam, #ab83366). Significance is indicated as *p=0.0117

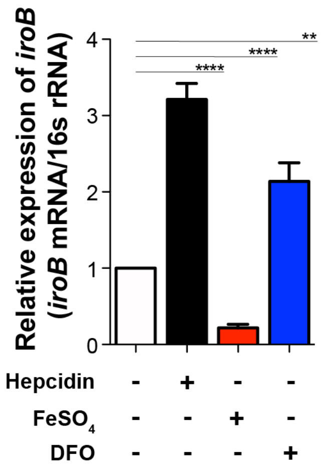

Supplementary Figure 9. Activity of *iroBp* in the *Salmonella* infecting Raw264.7 cells pretreated with PBS, hepcidin (1 μg/ml), $FeSO_4$ (0.1 mM), or DFO (100 μM), as determined by qPCR for *iroB* mRNA. Significance is indicated as **p=0.0012; ****p<0.0001

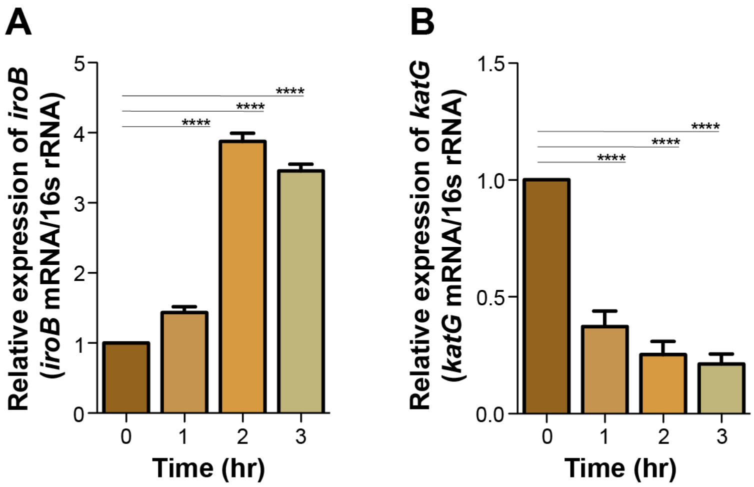

Supplementary Figure 10. Time-dependent changes in *iroBp* (A) and *katGp* (B) activity in the *Salmonella* infecting Raw264.7 cells pretreated with hepcidin, as determined by qPCR. Significance is indicated as ****p<0.0001

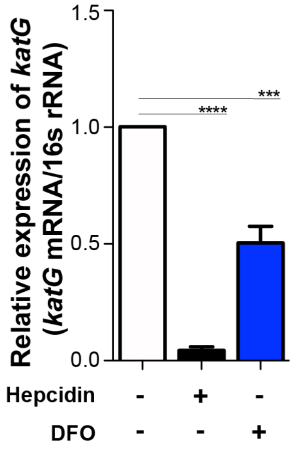

Supplementary Figure 11. The activity of *katGp* in the *Salmonella* infecting Raw264.7 cells pretreated with PBS, hepcidin (1 μg/ml), or DFO (100 μM) was determined by qPCR for the level of *katG* mRNA relative to that of 16S rRNA. Significance is indicated as ***p=0.0003; ****p<0.0001

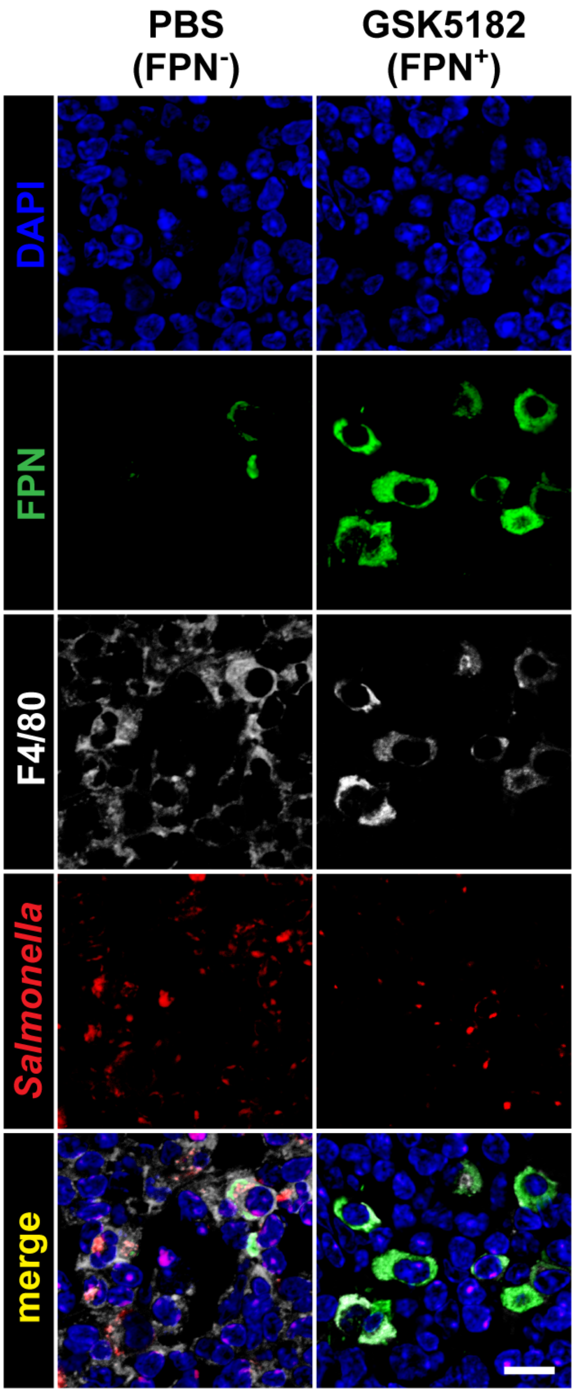

Supplementary Figure 12. Localization of FPN in the spleen of *Salmonella*-infected mouse. WT mice were infected with *Salmonella* and treated with GSK5182 or PBS as described in Figure 5 (n=5 per group). The spleens were isolated 2.5 days *p.i.* and examined for FPN by confocal microscopy using anti-FPN antibody. F4/80$^+$ macrophages were identified using anti-F4/80 antibody.

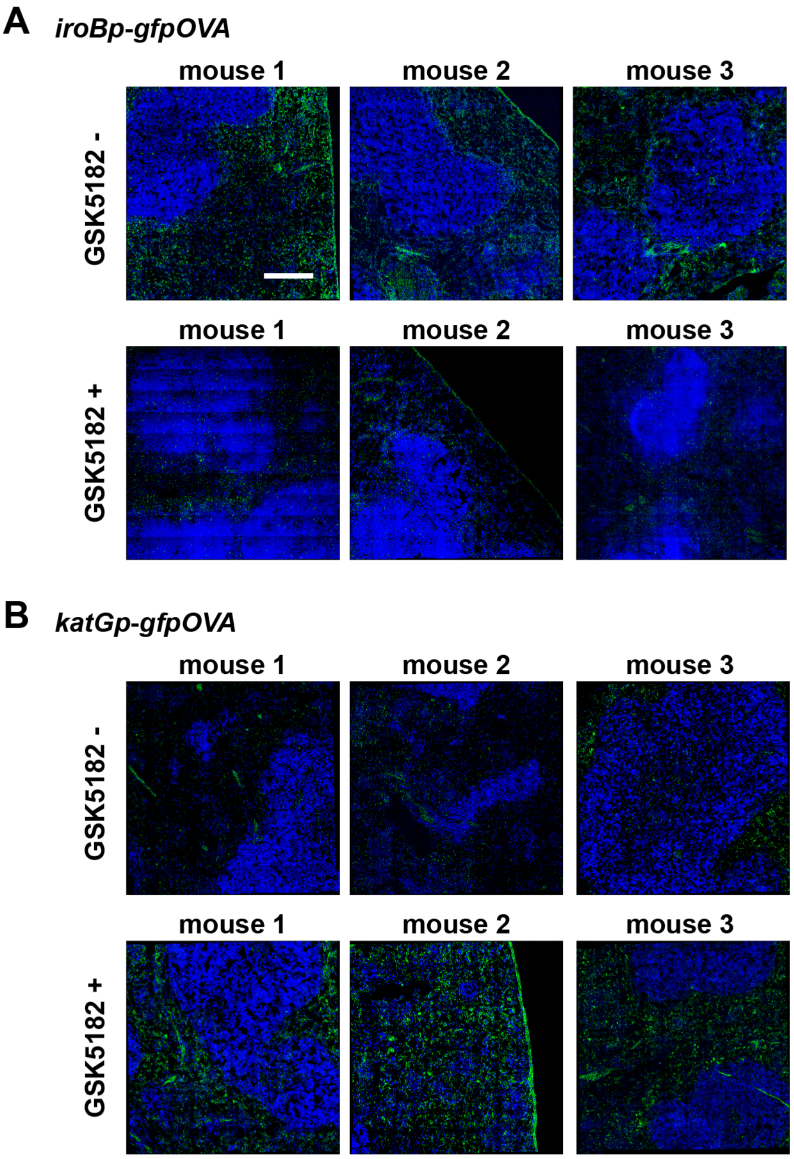

Supplementary Figure 13. The tile-scan-images (10x10 fields) of infected spleens shown in Figure 6A. WT mice were infected with *Salmonella* containing *iroBp-gfpOVA* or *katGp-gfpOVA* biosensors and treated with GSK5182 or PBS. The spleens were isolated 2.5 days *p.i.* and checked for *iroB* (A) or *katG* (B) expression by confocal microscopy. Scale bar, 200 μm.

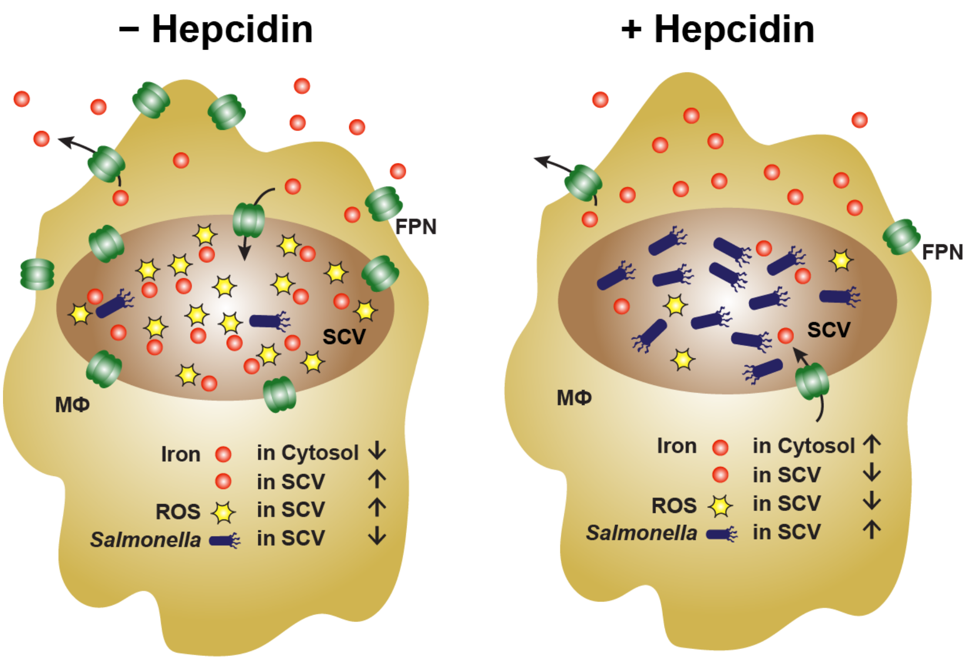

Supplementary Figure 14. Graphic Summary

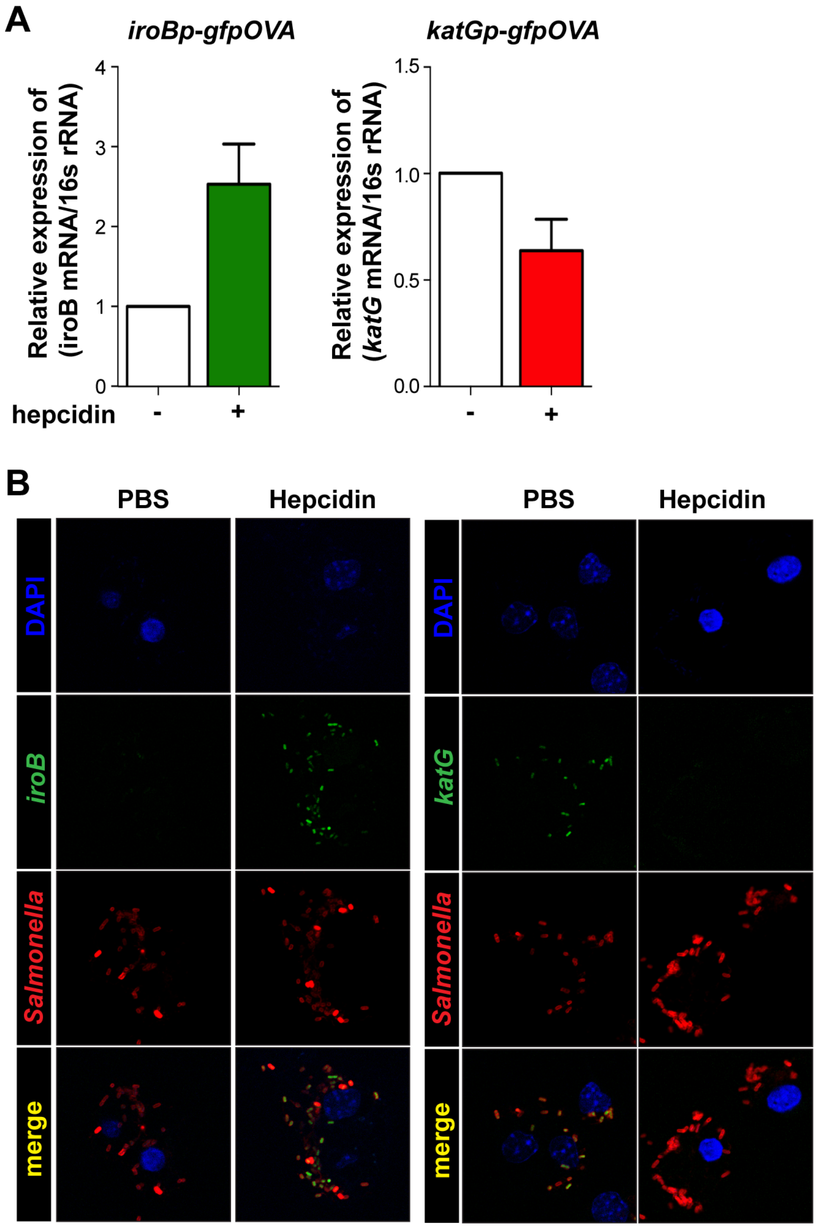

Supplementary Figure 15. The expression of *iroBp-gfpOVA* or *katGp-gfpOVA* in peritoneal macrophage isolated from DBA mice with or without hepcidin treatment (n=5 per group). Peritoneal macrophages were isolated from DBA (Nramp$^{+/+}$) mice, treated with PBS or hepcidin (1 μg/ml) and infected with *Salmonella* carrying episomal *iroBp-gfpOVA* or *katGp-gfpOVA* biosensors. The expression level was measured by qPCR for the level of *iroB* or *katG* mRNA relative to that of 16S rRNA. Significance for *iroB* is p=0.0003; for *katG*, p<0.0001 (A). The images are taken 10 min *p.i.* using a confocal microscope (B).

Figure 1C

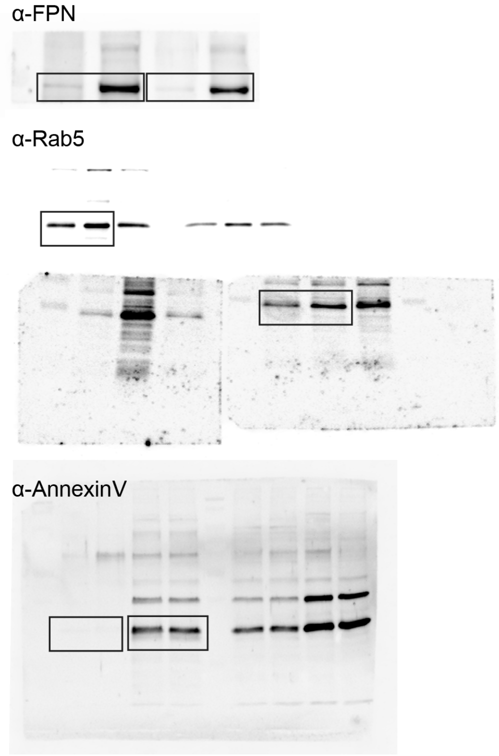

Figure 1D

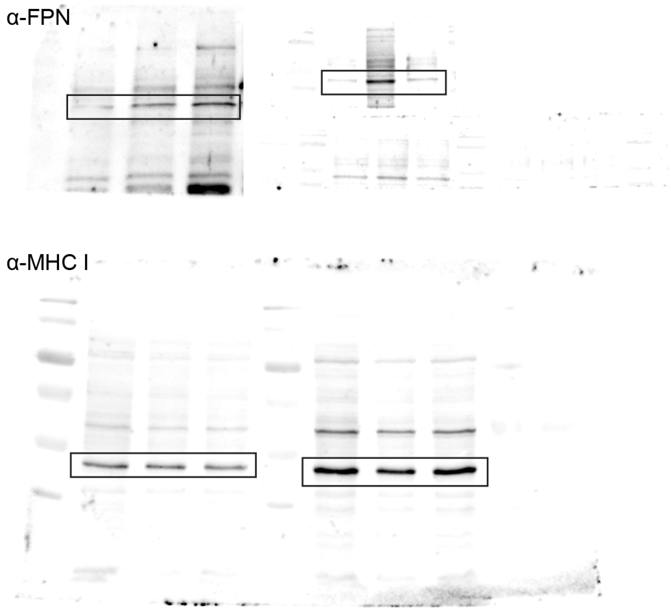

Supplementary Figure 16. Full images of western blot figures shown in the main paper.

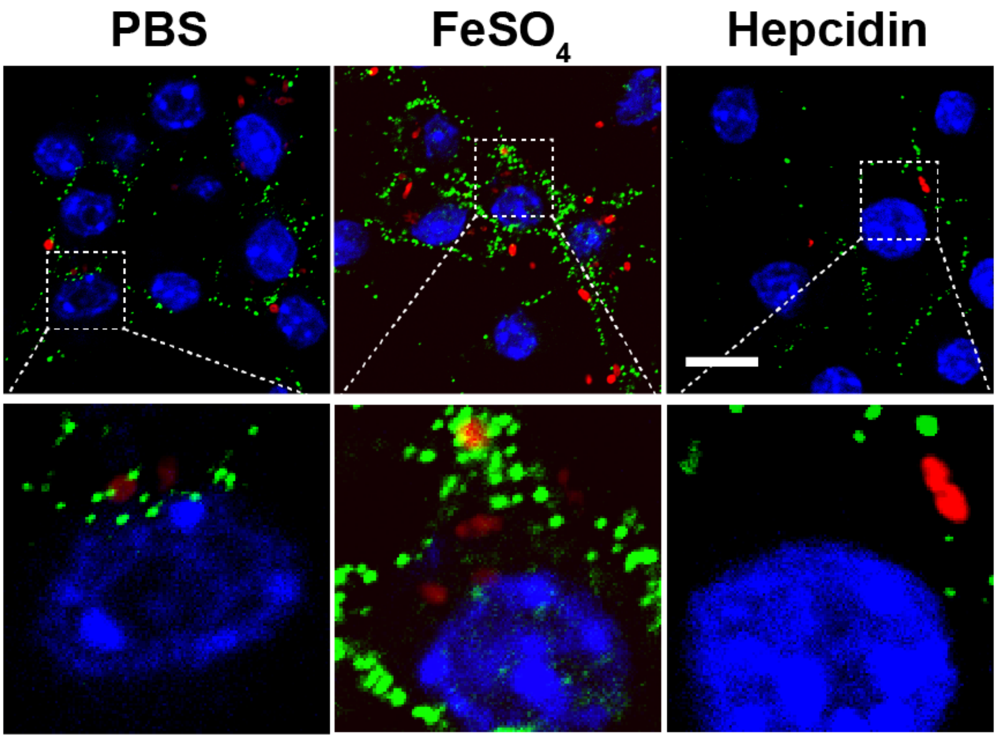

Supplementary Figure 17. Enlarged images of merge panels shown in Figure 1A.
